# Investigating the Experiences of Childhood Cancer Patients and Parents Participating in Optional Nontherapeutic Clinical Research Studies in the UK

**DOI:** 10.1002/pbc.25960

**Published:** 2016-03-01

**Authors:** Julie Errington, Ghada Malik, Julie Evans, Jenny Baston, Annie Parry, Lisa Price, Hina Johnstone, Selena Peters, Victoria Oram, Karen Howe, Emma Whiteley, Jane Tunnacliffe, Gareth J. Veal

**Affiliations:** ^1^ Northern Institute for Cancer ResearchNewcastle UniversityNewcastle upon TyneUK; ^2^ Pediatric Oncology and HaematologyLeeds General InfirmaryLeedsUK; ^3^ Clinical Trials UnitGreat North Children's HospitalNewcastle upon TyneUK; ^4^ Institute for Child Life and HealthBristol UniversityBristolUK; ^5^ Great Ormond Street HospitalLondonUK; ^6^ Royal Manchester Children's HospitalManchesterUK; ^7^ Addenbrooke's HospitalCambridgeUK

**Keywords:** clinical trials, parents, patient experiences, pediatrics, research

## Abstract

**Background:**

While the majority of childhood cancer clinical trials are treatment related, additional optional research investigations may be carried out that do not directly impact on treatment. It is essential that these studies are conducted ethically and that the experiences of families participating in these studies are as positive as possible.

**Methods:**

A questionnaire study was carried out to investigate the key factors that influence why families choose to participate in optional nontherapeutic research studies, the level of understanding of the trials involved, and the experiences of participation.

**Results:**

A total of 100 participants from six UK centers were studied; 77 parents, 10 patients >16 years, and 13 patients aged 8–15 years. Ninety‐seven percent of parents and 90% of patients felt that information provided prior to study consent was of the right length, with 52% of parents and 65% of patients fully understanding the information provided. Seventy‐four percent of parents participated in research studies in order to “do something important”, while 74% of patients participated “to help medical staff”. Encouragingly, <5% of participants felt that their clinical care would be negatively affected if they did not participate. Positive aspects of participation included a perception of increased attention from medical staff. Negative aspects included spending longer periods in hospital and the requirement for additional blood samples. Ninety‐six percent of parents and 87% of patients would participate in future studies.

**Conclusions:**

The study provides an insight into the views of childhood cancer patients and their parents participating in nontherapeutic clinical research studies. Overwhelmingly, the findings suggest that participation is seen as a positive experience.

**Abbreviations**
CCRNG: Childhood Cancer Research Nurses Group
COG: Children's Oncology Group
ISRCTN: International Standard Randomised Controlled Trial Number

## INTRODUCTION

It is estimated that approximately 60% of children diagnosed with cancer in the UK participate in clinical trials.^1^ The vast majority of these studies are therapeutic trials, involving the recruitment of patients to treatment protocols in order to answer specific questions about the drugs being tested and assess how effective they are in the treatment of various different types of childhood cancer. This has led to significant advances in the treatment of pediatric malignancies over the past several decades.^2^

Clinical trials in the modern era have advanced considerably in terms of their complexity, with numerous “substudies” commonly included in the trial design to learn as much as possible about the treatments being investigated. Although interventional, commonly involving the collection of additional clinical samples, these optional studies will not directly impact on the treatment that they receive. Similarly, in addition to therapeutic trials that largely define the treatment of the childhood cancer patient, there are also commonly stand‐alone nontherapeutic trials that do not provide treatment to patients, but may facilitate the generation of knowledge to help advance our understanding of cancer and its treatment. In these scenarios, where the patients will not benefit directly from their inclusion in the trial or substudy, ethical questions may be asked about the appropriateness of study participation for children with cancer. Potential concerns may include the negative impact of being separated from parents, concerns over the frequency and volume of clinical samples being collected for research purposes, in addition to discomfort and inconvenience brought about by study involvement. In this respect, our group and others have previously attempted to assess the impact of involvement in clinical studies involving the collection of blood samples for research purposes and carried out studies to help reduce the impact of such studies on the patients involved.^3^, ^4^, ^5^, ^6^

From a scientific perspective, it is fortunate that childhood cancer patients and their families are frequently willing to participate in nontherapeutic studies in order to advance our understanding of cancer and its treatment. However, poor experiences for patients may lead to high rates of study drop out or low recruitment rates, with a clear negative impact on achievement of the scientific aims of the study. In considering the future involvement of patients in clinical trials, it is important that we consider the experiences of families who have recently been involved in optional research studies and investigate the positive and negative experiences of participation. Previous work in this area includes studies assessing the views of parents on consent and participation in therapeutic trials for cancer and other illnesses,^7^, ^8^, ^9^, ^10^, ^11^ but very few studies have been published focusing on the views of both patients and parents of participation in nontherapeutic trials purely in an oncology setting.

The current study was designed to gain information relating to the key factors that influence why childhood cancer patients and their families choose to participate in optional clinical research studies, the level of understanding of the purpose of the clinical trial, and the pros and cons of participation. By learning more about the experiences of patients and families participating in these important studies, researchers and clinical staff can look to design and conduct trials in such a way as to improve practices and maximize recruitment to future studies.

## METHODS

### Study Design

Ethical approval for the study was gained from the Sunderland Research Ethics Committee. The questionnaire was devised following discussions with the Childhood Cancer Research Nurses Group (CCRNG), with approaches taken in previously published studies in this area taken into account to inform the initial structure of the questionnaire.[9] Two questionnaires were designed for use in the study, one for parents and patients >16 years of age and a simplified version for patients aged 8–16 years (see Supplementary Fig. S1). The questionnaire included predominantly structured questions, designed in conjunction with the CCRNG, with each question providing an additional free text option to ensure that participants did not feel limited by the questionnaire structure. A final questionnaire was approved following completion of a limited center pilot study involving 20 patients/families. The questionnaire was designed to cover the following key areas: experiences of the trial recruitment process, level of understanding of the information provided leading to study consent, and experiences of the clinical research study day. Table I provides a summary of key questions included in the questionnaire, for each of which there were 2–4 graded answers provided for the participants to select as appropriate. The content and construct validity of the questionnaire was not examined.

**Table I:** Summary of Questions Included in the Study Questionnaire

Were you happy with the information you were given before you consented to the study?
Do you think that the parent/patient information sheet was of the appropriate length?
Do you think that the parent/patient information sheet was understandable?
How well did you understand the purpose of the trial?
Why did you decide to take part in the study?
Did you feel that making an extra visit to the hospital on the research study day was a chore?
Did you feel that the length of time the study took was too long?
Did you feel that the number of blood samples taken for the research study was too many?
In your opinion what were the positive aspects of taking part in the research study?
In your opinion what were the negative aspects of taking part in the research study?
Did you feel that you were approached at the right time about taking part in the research study?
Would you/would you allow your child to take part in future research studies?

### Recruitment

The eligible study population included childhood cancer patients and their parents, who had participated in a nontherapeutic clinical trial between March, 2010 and May, 2014. It was stipulated that questionnaires should be completed within a 12‐month period following participation in the clinical research trial. Patients ≥8 years old were invited to complete an age appropriate questionnaire themselves, with parents asked to complete the questionnaire for patients <8 years and older patients who did not wish to complete an independent questionnaire.

### Data Collection

Families were approached by the research nurses at a clinic follow‐up visit or during their hospital stay and invited to participate. Questionnaires were completed by patients or parents during the clinic visit, collected by research nurses at the end of the visit, and sent to Newcastle for data analysis. Informed consent or assent as appropriate was taken from all study participants prior to completion of age‐appropriate questionnaires. Demographic details of the individuals completing the questionnaire were not collected, with the exception of age for patients completing the questionnaire.

### Analysis of Questionnaire Data

The results obtained from the questionnaire were grouped into four basic areas of interest: “level of understanding of research study aims,” “reasons for participation in clinical research study,” “impact of study participation,” and “positive and negative aspects of participation.” In addition to the presentation of numerical data obtained from the questionnaires, free text comments provided by participants were also considered.

### Statistical Analysis

Based on data generated from a pilot study of 20 patients/families, a total of 100 patients were included in the final study proposal. Inclusion of 100 patients provided a 90% probability that the width of the 95% confidence interval for the estimated proportions (comparable data to the responses generated in the pilot study) would be less than 0.14 (95% confidence intervals of [0.83, 0.97] and [0.03, 0.17]). This was seen as an acceptable level of precision for the proposed study design. A formal statistical analysis was not carried out on the results obtained from the study.

## RESULTS

### Patient Accrual

A total of 100 participants were recruited to the study, 77 parents, 10 patients >16 years old, and 13 patients aged 8–15 years. All patients had participated in nontherapeutic clinical research studies no longer than 12 months prior to completion of the questionnaire. Patients had participated in the following nontherapeutic trials or nontherapeutic substudies within larger clinical trials (ClinicalTrials.gov or International Standard Randomised Controlled Trial Number [ISRCTN] identifiers: NCT00897871, NCT00900354, NCT00939965, NCT01704716, ISRCTN64515327, and ISRCTN52616678). All of these studies were comparable in terms of requesting blood samples at specific times during treatment for analysis of drug levels (pharmacokinetic studies) in addition to a blood sample collected pretreatment for pharmacogenetic analysis. Participants were recruited from six clinical centers involved in the treatment of children with cancer across the UK.

### Level of Understanding of Research Study Aims

**Figure 1:**
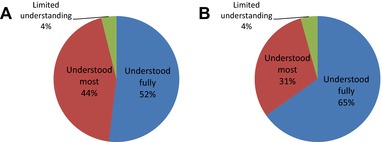
Views of parents (**A**) and patients (**B**) on the level of understanding of the purpose of the nontherapeutic clinical research study in which they participated.

In terms of the information that was provided prior to participation in a nontherapeutic research study in the form of parent or patient information sheets, 97% of parents and 90% of patients felt that the information provided was of the right length, with the remaining participants finding information sheets to be too long. Approximately half of the parents (52%) felt that they fully understood the information provided, as compared to 65% of patients, with 44% of parents and 30% of patients understanding the majority of information provided (Figs. 1A and 1B). It should be noted that for patients recruited to nontherapeutic research studies, information would have been provided in the form of age‐appropriate information sheets (<8 years old, 8–12 years, 13–15 years, and ≥16 years). There was no clear relationship apparent between the level of understanding stated and the age of the patient studied, although the study size was small and no statistical analysis was carried out. The vast majority of participants (>99% of both parents and patients) felt that they were approached about participating in the study at the right time.

### Reasons for Participation in Clinical Research Study

Table II provides information relating to the reasons for participating in nontherapeutic clinical research studies. For the majority of parents, altruistic motives were clearly important factors, with 74% of parents expressing the “feeling of doing something important” as a key reason for participating and 53% of parents feeling that they were “helping medical staff/giving something back”. These figures compared to values of 26 and 74%, respectively, for responses obtained from patients to the same questions. Encouragingly, less than 5% of responders felt that their clinical care would be affected if they did not participate or that they were in any way obliged to participate in nontherapeutic clinical research studies.

**Table II:** Reasons for Participating in Nontherapeutic Clinical Research Studies

	Number of respondents	
Reasons for participating	Parents (%)	Patients (%)
Felt obliged to participate	1 (1.3)	2 (9)
Felt that clinical care would be affected	2 (2.6)	0 (0)
Feeling of doing something important	57 (74)	6 (26)
Helping medical staff/giving something back	41 (53)	17 (74)

Participants were allowed to select as many reasons as they felt were appropriate.

### Impact of Study Participation

Answers to questions relating to the impact of being required to make extra visits to hospital and the length of the study day were complicated by the fact that some patients and parents were not required to attend extra visits as they were being treated as inpatients. In this scenario, the impact of the additional research study would have largely been in relation to the provision of additional clinical samples outside of what would have been collected as part of the routine care of the patient. For those patients who were required to make additional visits to the hospital for the research study, 23 of 24 (96%) parents who responded were happy to make the extra visit as compared to five of nine (56%) patients. No parents or patients felt that the research study day was too long.

As patients and parents completing the questionnaire had participated in clinical pharmacology studies, involving the collection of sequential blood samples over a defined time period, a key question was included within the questionnaire related to the number of blood samples being collected for research purposes. Interestingly, only 8% of parents and 9% of patients indicated that too many blood samples were being taken as part of the research study when this question was directly asked within the questionnaire.

### Positive and Negative Aspects of Participation

**Figure 2:**
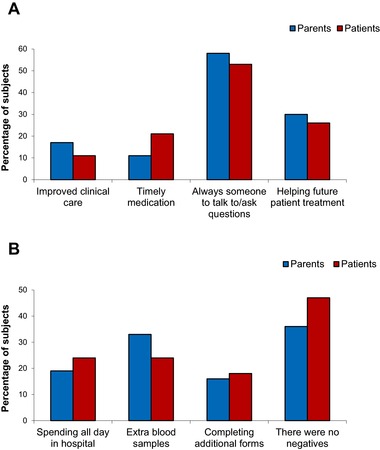
Views of parents and patients on the positive (**A**) and negative (**B**) aspects of participating in nontherapeutic clinical research studies.

Responses to the question of what were deemed to be the positive aspects of taking part in the research study were in alignment with the altruistic reasons for participating in the studies, with 30% of parents and 26% of patients stating this as a positive aspect of their involvement. Having a member of the clinical team available to talk to was given as a positive factor by over half of all respondents (58% of parents and 53% of patients), with a perceived improvement in clinical care and more timely administration of medication also highlighted. Factors that were seen as negative aspects of study participation included the collection of additional blood samples (33% of parents and 24% of patients), the need to spend longer periods in hospital (19% of parents and 24% of patients), and the requirement to read and sign additional forms (16% of parents and 18% of patients). Figures 2A and 2B summarize the positive and negative aspects of participating in clinical research studies, respectively, from perspectives of both parent and patient. The vast majority of parents (96%) and patients (87%) stated that they would participate in future clinical research studies if asked. There was no indication from the results obtained for any apparent trend between responses and age of participant, although no formal statistical analysis was carried out.

## DISCUSSION

Bearing in mind the challenges of recruiting patients to pediatric clinical trials, it is clearly important to understand why parents consent to their children participating in these studies. While many studies have previously investigated the views of patients and parents in relation to the provision of consent and participation in clinical trials across a wide range of disease types, the majority of these studies related to therapeutic clinical trials involving treatment that may benefit the child being studied. Indeed, the potential for clinical benefit has previously been highlighted as a key factor influencing a parent's decision to provide consent in several studies both in the UK and other countries.^7^, ^9^, ^12^, ^13^ Even in a phase I trial setting, it has been widely reported that misconceptions regarding the potential for therapeutic benefit commonly influence decisions made regarding trial participation.^14^, ^15^ The current study was designed to gain information specifically relating to the factors that influence why childhood cancer patients and their families choose to participate in optional nontherapeutic clinical research studies, the level of understanding of the purpose of the clinical trial involved, and the pros and cons of participation.

When designing clinical trials that will not directly impact on the treatment of the children participating, it is essential that appropriate information is provided to ensure that the patients and parents providing consent understand exactly what is involved. In order to ensure that all children are aware of the reasons for carrying out the study and what participation entails, information sheets are routinely provided for children of varying ages and intellectual capacity. In this respect, the findings of the current study, in terms of level of understanding of the information provided, are encouraging. Although no formal testing of how well participants understood the trials to which they had been recruited, over half of the parents and two‐thirds of patients felt that they fully understood the information provided, with over 95% of participants understanding at least the majority of the information included in the study information sheets. While this was not formally addressed as part of the questionnaire, this would indicate a good understanding of what level of detail is required to be included in patient information sheets across a number of research studies to which patients were recruited. This is an area that has been successfully progressed over a number of years, supported by a trend toward obtaining input from patient advocacy groups when devising information sheets for clinical trials.^16^, ^17^ Similarly reassuring is the finding that families were happy with the amount of information provided and the timing that the research study was discussed with them. This can often be challenging in terms of not wanting to overload patients and parents with information at a difficult time, particularly in situations where studies are discussed relatively soon after cancer diagnosis.

As has been reported in previously published studies, a major factor that influenced study participation relates to the desire to help generate information that may lead to improved treatments for future childhood cancer patients. Although many previous studies have reflected on results obtained from single studies,^7^, ^9^ and therefore may not have been generalizable to other studies with different risk/benefit profiles, our data have been generated from patients participating in six different clinical trials. These studies included both stand‐alone clinical research trials and optional nontherapeutic substudies incorporated in larger national trials. Despite the relatively high‐risk nature of the cancers involved across these studies, the level of altruism exhibited is clearly a common factor influencing the level of participation. Comments provided by parents such as “I was delighted we could do something vaguely useful and wish we could have been part of more studies” and “I am pleased to be able to help as the findings may benefit future parents in my current situation” were frequently included in the free text sections of the questionnaire forms returned. Additional factors cited as important reasons for participation included a desire to want to help medical staff conducting clinical research trials and wanting to give something back to the clinical team involved in their treatment. A particularly reassuring finding from the current study is that very few participants felt any kind of obligation or pressure to participate or that their clinical care may be affected if they did not consent. This finding underlines the importance of providing families with information sheets that very clearly delineate between treatment and research trial procedures.

The findings of the current study should be looked at in the context of a similar study carried out by the Children's Oncology Group (COG) in the United States.^18^ The COG study enrolled 36 patients into a study investigating the reasons for participating in optional pharmacokinetic studies incorporated into phase 1 studies in a childhood cancer setting. While there are a number of similarities between these two studies, in terms of the patient population and the requirement for the collection of multiple blood samples at defined times following drug administration, some interesting differences are observed in the results obtained. A factor that was highlighted in the U.S. study as being a concern to the parents and patients studied was the requirement for additional blood samples that may expose the patient to additional risks and potential discomfort. Similar findings have been reported from previous studies, with 42% of patients stipulating blood procurement as a reason for nonenrollment into a controlled vaccine trial.^19^ While this was also a negative aspect of study participation in the current UK study, with approximately one‐third of participants selecting the collection of extra blood samples as a negative aspect of study participation, only 8% of parents and 9% of patients felt that too many blood samples were taken. Indeed, several parents commented that the number of samples collected for research purposes was trivial in relation to the total number collected for routine clinical care. This raises two important points. First, it is important to note that although patients in the COG study had IV catheters inserted for blood sampling as part of the pharmacokinetic study, it was stipulated in all of the UK research studies onto which patients were recruited that all samples were taken from central lines. Indeed, the U.S. study highlighted a need to validate methods that permit samples to be drawn from indwelling central lines, an approach that has been in place in UK pediatric oncology centers for ethical reasons for many years. Second, it should be highlighted that information sheets used for UK research studies in a pediatric setting frequently include a very clear statement concerning the maximum volume of blood to be taken and the potential impact on the child participating in the study. Again, this underlines the importance of providing clear information for patients and parents prior to their recruitment into a clinical research study.

In summary, we can be encouraged that the results from the current study indicate that we are running well‐planned and informative clinical research studies, with patients and parents expressing positive views on their experiences following participation. However, we also need to be aware of the limitations of the study carried out. These include the relatively limited number of children directly participating in the study and the fact that the impact of study participation is likely to have been variable depending on whether patients were being treated as inpatients or outpatients. Similarly, it may have been informative to collate additional information during the study, including the number of potential participants who were approached about the study but chose not to participate and additional patient demographics including the clinical outcome of the patients on the clinical trials in which they participated.

Overall, the take‐home messages from the current study are clearly positive and the findings bode well for future trial recruitment, which is essential to make further advances in the treatment of children with cancer. As one parent of a child being treated for high‐risk neuroblastoma commented, “we would be more than happy to participate in any future studies as I believe this is a huge part of finding out more about curing this disease”. Sharing the findings from this study with parents and families considering participation in future nontherapeutic research studies will support the recent provision of excellent material currently available through the Nuffield council.^20^ Future studies of this nature may benefit from focusing on obtaining the views of an increased number of children participating in nontherapeutic research studies, as opposed to parents, in addition to addressing some of the limitations of the current study outlined above.

## Supporting information

Supplementary Fig. S1. Questionnaires utilised in the study for parents and patients 16 years and over and for patients under 16 years.
