# Supplementary material for: Investigating the Experiences of Childhood Cancer Patients and Parents Participating in Optional Nontherapeutic Clinical Research Studies in the UK
Source: Pediatr Blood Cancer. 2016 Mar 1;63(7):1193–7. doi: 10.1002/pbc.25960 (PMC5031198; doi:10.1002/pbc.25960)
Supplement: Supplementary file 1 — Supplementary Fig. S1. Questionnaires utilised in the study for parents and patients 16 years and over and for patients under 16 years. [file PBC-63-1193-s001.pdf]

Supplementary Figure 1.

A study to investigate the experiences of families participating in paediatric pharmacology cancer trials.

(NICR/2010/01)

Version 2, 1st February 2012

Questionnaire for parents & patients 16 years and over

Study ID : ____/____<br>(Centre abbreviation/study number)<br><br>**To be filled in by the research nurse only**

Thank you for agreeing to participate in this study.

These questions relate to your participation in one of the CCLG Pharmacology Group studies.

There are no right or wrong answers. If you do not understand a question, please ask for help. **Please tick all boxes that apply**.

For most questions there will be space to write your own comments, please feel free to be as critical or positive as you wish.

I am : The patient ☐ The parent ☐

The pharmacology study I / my child took part in was;

_______________________________________ (please enter)

e.g. PK 2008 03

1. Were you happy with the information you were given before you consented to the study? Yes ☐ No ☐

Comment:

2. Did you think that the parent / patient information sheet was?

Too long? ☐ The right length? ☐

Too short? ☐

Comment:

3. Did you think that the parent / patient information sheet was?

Too complicated? ☐ Understandable? ☐

Too simple? ☐

Comment:

4. How well did you understand the purpose of the study?

Fully	☐	Limited understanding	☐
Well enough	☐	No understanding	☐

Comment:

5. Why did you decide to take part in the study?

- I felt I/we had to ☐
- I felt that my/my child's treatment would be affected if I didn't take part ☐
- I felt like I was/we were doing something really important ☐
- I wanted to help the doctors because they helped me/my child ☐

Any other reasons?

6. On the study day;

- I was happy to come in for an extra visit ☐ N/A ☐
- I felt that having to come for an extra visit was a chore ☐

Comment:

7. Did you feel that the length of time the study took was;

Far too long ☐ Long but bearable ☐ OK ☐

Comment:

8. Did you feel the number of blood samples taken for the study was;

Too many ☐ Not a concern for me ☐

Comment:

9. The positive aspects of taking part in the study were;

- I felt that I /my child got lots of attention ☐
- I/my child got all of my/their medicines on time ☐
- I felt that there was always someone around to talk too/ask questions ☐

Other reasons:

10. The negative aspects of taking part in the study were;

- Having to spend all day in hospital ☐
- Having to have extra blood samples taken ☐
- Reading / filling in additional forms ☐

Other reasons:

11. I would/I would allow my child to take part in a pharmacology study again;

Yes ☐ No ☐

12. Did you feel you were approached about taking part in the study;

- At the right time ☐
- Too long before the study took place ☐
- It could have been earlier ☐

Thank you for completing this questionnaire.

Please hand it back to your research nurse before you leave today

A study to investigate the experiences of families participating in paediatric pharmacology cancer trials.

(NICR/2010/01)

Version 2, 1st February 2012

Questionnaire for patients Under 16 years

Study ID : ____/____
(Centre abbreviation/study number)
**To be filled in by the research nurse only**

These questions are about when you took part in one of the CCLG Pharmacology Group studies.

There are no right or wrong answers. If you do not understand a question, please ask for help.

Please tick all boxes that you agree with. There is also a space under some of the questions to write any extra information you think might help us.

I am ______ years old.

The pharmacology study I took part in was;

____

(please ask your parents or your research nurse if you don't know)

1. Were you happy with the information you were given before you decided to take part in the study?

Yes ☐ No ☐

2. What did you think about how long the patient information sheet was?

It was too long ☐ It was the right length ☐

It was too short ☐

3. How much could you understand from the patient information sheet that you were given?

I could understand it ☐ It was too complicated ☐

It was too simple ☐

4. How well did you feel you understood what the study was about?

I understood it ☐ I understood most of it ☐

Only a little bit ☐ I didn't understand it at all ☐

5. Why did you want to take part in the study?

I felt I had to ☐

I felt that I wouldn't get my medicine if I didn't take part ☐

I felt like I was doing something really important ☐

I wanted to help the doctors because they helped me ☐

Any other reason?

6. On the study day;

I didn't mind having to go to the hospital to take part in the study ☐

I was not happy to go to the hospital to take part in the study ☐

I was already in hospital ☐

7. Did you think that the study day was;

Far too long ☐ Too long ☐ OK ☐

8. Did you feel the number of blood samples taken was;

Too many ☐ OK ☐

9. The best reasons for taking part in the study were;

- I felt that I got lots of attention ☐
- I got all of my medicines on time ☐
- There was always someone to talk to/ask questions ☐

Any other reasons?

10. The parts of the study I didn't like were;

- Having to spend all day in hospital ☐
- Having to have extra blood samples taken ☐

Any others?

11. I would take part in a pharmacology study again;

Yes ☐ No ☐

Thank you for completing this questionnaire.

Please hand it back to your research nurse before you leave today
